# Mechanism Study on the Influence of Clay-Type Lithium Slag on the Properties of Cement-Based Materials

**DOI:** 10.3390/ma18081788

**Published:** 2025-04-14

**Authors:** Kejia Xiao, Guangshao Yang, Wei Zhou, Qihao Ran, Xin Yao, Rengui Xiao, Shaoqi Zhou

**Affiliations:** 1 School of Resources and Environment, Guizhou University, Guiyang 550025, China; xiao_kejia@163.com; 2 School of Chemistry and Chemical Engineering, Guizhou University, Guiyang 550025, China; 15186002564@163.com (G.Y.); 13765671705@139.com (Q.R.); rgxiao@gzu.edu.cn (R.X.); 3 School of Modern Agricultural, Yiyang Vocational & Technical College, YingFeng Qiao, Yiyang 440100, China; 17507376635@163.com; 4 The Third Surveying and Mapping Institute of Guizhou Province, Guiyang 550025, China; venus124127@126.com

**Keywords:** clay-type lithium slag, hydration kinetics, hydration products, microstructure

## Abstract

With the increasing demand for lithium resources and the enhancement of global environmental awareness, how to efficiently and environmentally develop clay-type lithium resources is of great strategic significance for future development. Clay-type lithium slag (LS) is a byproduct resulting from the extraction of lithium from clay-type lithium ores. Its primary chemical constituents include SiO_2_ and Al_2_O_3_, and it exhibits potential pozzolanic properties. Clay-type lithium ore is of low grade, so a large amount of clay-type LS is produced during its production. In this study, calcined clay-type LS, limestone powder (LP), and cement clinker were used as the main raw materials to prepare low-carbon LC^3^ cementitious materials. The study focused on the effect of clay-type LS and LP on the new mixing properties, mechanical properties, hydration kinetics, and microstructure formation and transformation of the cementitious materials. The findings revealed that incorporating clay-type LS and LP significantly raised the standard consistency water demand of cement and reduced the setting time of the binding material. While clay-type LS and LP initially weakened the mechanical performance of the cement mortar, it enhanced these properties in the later stages. The compressive strength of LC-10 and LC-20 at 180 days exceeded that of the reference by 3.7% and 1.1%, respectively. In addition, the number of micropores between 3 and 20 nm in LC^3^ cement increased significantly. It showed that the addition of clay-type LS and LP could optimize the pore structure to some extent. According to research, the optimal content of clay-type LS and LP should not exceed 30%. This method not only consumes the solid waste of clay-type LS, but also facilitates the green and low-carbon transformation of the cement industry.

## 1. Introduction

Lithium is an important energy material [1,2]. The lithium reserves of China are 6.8 million tons, ranking fifth in the world [3]. Naturally occurring lithium resources are primarily categorized into three types: brine, pegmatite, and clay, representing approximately 64%, 29%, and 7% of the total reserves, respectively [4]. Different kinds of lithium resources have different phases. Brine-type lithium resources mainly exist in salt-lake brine in the form of dissolved lithium salt. In addition to lithium, salt-lake brine has a complex composition and is also rich in other salts such as magnesium, sodium, and potassium [5,6]. Pegmatitic-type lithium ore mainly refers to spodumene, diatherite, and so on. Among these, spodumene belongs to the monoclinic pyroxene group and has a chain silicate structure. The main components of pegmatitic lithium ore are SiO_2_, Al_2_O_3_, Li_2_O, and other metal oxides [1]. The main clay minerals containing lithium are lepidoliteand lithium montmorillonite [7]. The clay minerals containing lithium belong to phyllosilicates. The crystal structure is mainly composed of two tetrahedral sheets and one octahedral sheet [8]. Currently, studies on the extraction and utilization of lithium resources, both domestically and internationally, predominantly concentrate on brine- and pegmatite-based lithium deposits [9,10]. However, due to the depletion of lithium ores and the high cost of solvents used to separate lithium from salt-lake brine, clay-type lithium ores are receiving increasing attention.

Due to the low grade of clay-type lithium ore, the extraction process generates significant amounts of residual waste. If the clay-type lithium waste residue is not properly treated, it not only will occupy a large amount of land resources, but also pose the risk of environmental pollution [11,12,13]. In addition, it will gradually accumulate in the human body according to the food chain, causing harm to life and health [14,15,16]. Therefore, it is urgent to treat the clay-type LS. At present, the treatment methods of LS are valuable component recovery [17], tailings made into building materials [18,19,20,21,22], and backfilling gob [23,24,25]. Among these, building materials are one of the important ways to realize the recycling of lithium waste residue.

Supplementary cementing materials (SCMs) are materials that partially replace Portland cement in cement or concrete to improve the performance of concrete or reduce the environmental impact of cement production. The commonly used SCMs mainly include fly ash, blast furnace slag, silica fume, metakaolin, and so on, which can be used in combination [26,27]. Generally, SCMs have a filling effect, dilution effect, and nucleation effect [28,29]. However, clay-type LS exhibits inertness at room temperature. Before clay-type LS can serve as an SCM in cement, its pozzolanic reactivity must be activated [30,31,32,33,34,35]. Under normal circumstances, there are three methods to stimulate the activity of the volcanic ash in clay-type LS, which are mechanical (physical) excitation, chemical excitation, and high-temperature excitation [36]. Mechanical (physical) excitation usually refers to the use of physical crushing or grinding methods to improve the fineness of particles. This method is mainly to cause different degrees of dislocation defects and recrystallization in the crystal lattice of the minerals [37,38]. Chemical activation involves enhancing the dissolution rate of the glassy surface through the addition of acidic, alkaline, or sulfate-based activators [39]. Thermal activation is the process of heating that leads to the dehydroxylation of mineral structures, resulting in structural fractures that enhance the chemical properties of the material [40]. Liu et al. [41] used a combination of high-temperature activation and chemical activation to improve the reactivity of LS. High-temperature calcination enhances the reactivity of LS, significantly increasing its active amorphous content from 17.3% to 80.7%. In addition, more active ingredients in LS increase the rate and degree of reaction. He et al. [42] conducted calcification and thermal activation treatment of LS and analyzed its activation mechanism. The results showed that the hydration heat release peak, total hydration heat, and hydration products of LS increased significantly after calcification and thermal activation. After hardening, the matrix was denser and the porosity was lower.

To date, many studies have reported the effect of LS as SCMs on cement properties. The addition of LS can make up for the negative effect of cement dilution [43,44,45]. LS can play a chemical filling role, mainly in the micro-aggregate effect and volcanic ash effect, so as to effectively improve the microstructure [46,47,48]. LS particles fill the spaces between the cement particles, increasing the material’s compactness, thereby increasing its strength and durability [49]. When LS is incorporated into mortar, it interacts with the calcium hydroxide generated during cement hydration, producing additional C-(A)-S-H phases [21,50]. By producing more hydration products, the compressive strength, freezing resistance, chloride ion permeability resistance, elastic modulus, dry shrinkage rate, and creep property of mortar can be improved [51,52,53,54].

In order to improve the mechanical properties and microstructure of cement, it was decided to use calcined clay–limestone powder (LP) as the reference cement with three synergistic effects to produce low-carbon cement, called LC^3^ cement. LC^3^ cement can reduce CO_2_ emissions by 30–40% compared with conventional cement while significantly reducing energy consumption [55,56]. The fineness of calcined clay is smaller than that of cement clinker, so LC^3^ cement has a higher specific surface area than other cements. LP can be used to adjust the particle size distribution of the cement components to improve the workability and early strength of the cement. In addition, LP promotes hydration of the clinker by providing a suitable surface for the nucleation of the hydrate (packing effect) and facilitates hydration reactions in the presence of aluminates [57]. The synergy between the three allows for a higher clinker substitution rate and a denser microstructure, resulting in better mechanical properties and durability [58,59].

In this paper, low-carbon LC^3^ cement composed of clay-type LS, LP, and reference cement is studied. Macroscopic properties of the samples are analyzed according to mechanical properties, heat of hydration, standard consistency and setting time. Mineral phase evolution of the samples is analyzed by X-ray diffraction (XRD), thermogravimetric analysis (TG), and nuclear magnetic resonance (NMR). The microstructure of the samples is analyzed using scanning electron microscopy (SEM) and mercury intrusion porosimetry (MIP). The purpose of this paper is to realize the comprehensive utilization of clay-type lithium slag and provide a theoretical basis for comprehensive application.

## 2. Materials and Methods

### 2.1. Materials

**Figure 1:**
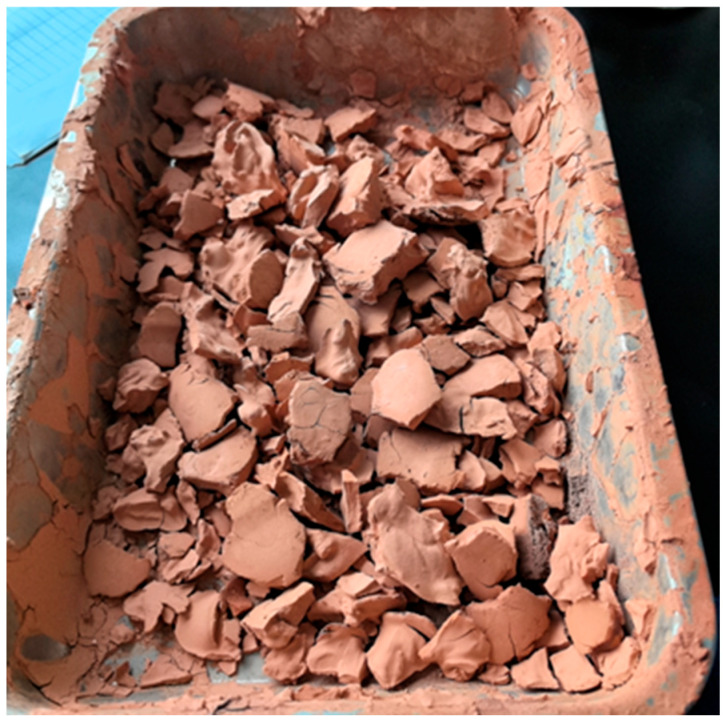
The morphology of LS.

Cement I, clay-type LS, and LP are utilized as binder materials. The appearance of clay-type LS is reddish brown, as shown in Figure 1. The chemical composition of raw materials is determined by X-ray fluorescence (XRF, Malvern Panalytical, Malvern, Britain). The chemical compositions are presented in Table 1. The main components of LS are SiO_2_ and Al_2_O_3_, and the total content of both reaches 93.13%, of which the content of SiO_2_ is as high as 73.53%. The mineral phases of Cement I include Tricalcium Silicate (C_3_S), Dicalcium Silicate (C_2_S), Tricalcium Aluminate (C_3_A), and Tetralcium Ferroaluminate (C_4_AF). The mineral phase of limestone powder is Calcite. The bulk densities of Cement I, clay-type LS, and LP were 3.04 g/cm^3^, 2.74 g/cm^3^, and 2.47 g/cm^3^, respectively.

### 2.2. Experimental Methodology

#### 2.2.1. Sample Preparation

Through previous studies, the method of high-temperature calcination was selected to stimulate the pozzolanic activity of clay-type LS. The optimum calcination system was determined as a heating time of 90 min; calcination temperature 900 °C; holding time 30 min; chill.

**Figure 2:**
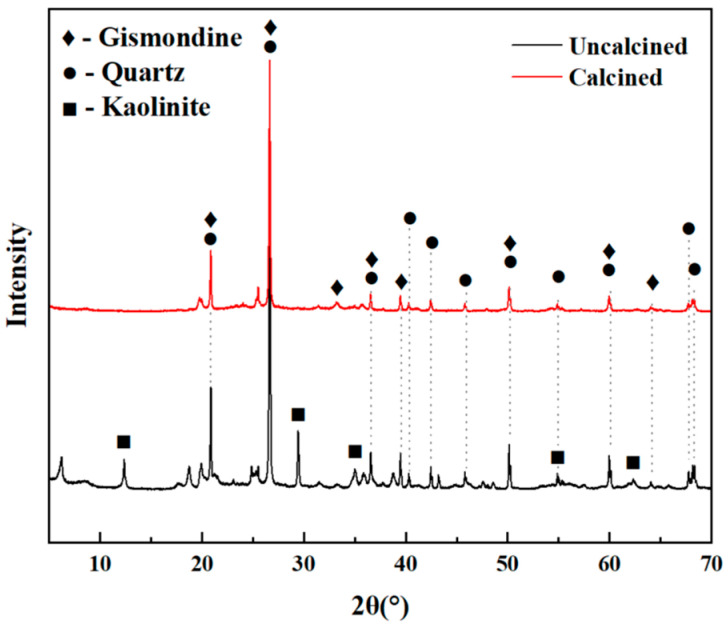
Mineral composition of uncalcined LS and calcined LS.

**Figure 3:**
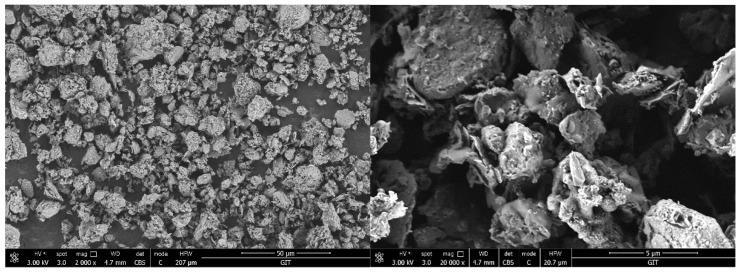
SEM images (2000× and 20,000×) of calcined LS.

XRD (D8 Advance diffractometer, Bruker Spectral Instruments, Beijing, China) was used to analyze the LS before and after thermal activation, and the results are shown in Figure 2. The primary mineral phases identified in clay-type LS were kaolinite, quartz, and gismondine. After high-temperature calcination, the characteristic peaks of kaolinite disappeared [60]. The diffraction peak of about 55° was formed by overlapping kaolinite peaks and quartz peaks, which were very close to each other. After calcination, the kaolinite peak disappeared, making the position of quartz more obvious in the XRD pattern of the calcined sample. As shown in the figure, kaolinite had undergone structural transformation. Thus, the volcanic ash activity of clay-type LS was activated. SEM (N.sem450 scanning electron microscope, FEI Corporation, Hillsboro, OR, USA) was used to characterize the microstructure of clay-type LS after thermal activation, as shown in Figure 3. The results indicated a porous, layered morphology in the material.

**Figure 4:**
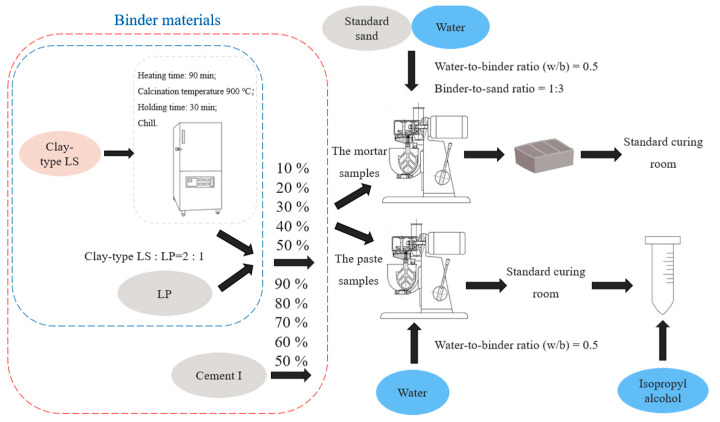
Flow chart of sample preparation.

The mortar specimens were prepared according to the GB/T-17671-2021 [61] under the conditions of water-to-binder ratio (w/b) of 0.5 and binder-to-sand ratio of 1:3. The recipes of mortar are displayed in Table 2. Among them, the sand was made of China ISO standard sand (Xiamen ASIO Standard Sand Co., Ltd., Xiamen, Fujian). China ISO standard sand meets both ISO679-2009 [62] and GSB 08-1337-2017 [63] standards. The effects of clay-type LS and LP on cement materials were studied by adding different amounts of clay-type LS and LP. The flow chart of sample preparation is shown in Figure 4. After completed mixing, the mortar samples were poured into a mold with a size of 40 mm × 40 mm × 160 mm. The mortar samples were formed on a vibrating table and cured at room temperature on the first day. The mortar samples were demolded and sent to the standard curing room (relative humidity > 80%, 20 ± 1 °C) after 24 h, and the samples were cured for 3 days, 7 days, 28 days, 60 days, and 180 days.

#### 2.2.2. Standard Consistency and Setting Time

The standard consistency water demand and setting times for reference and LC samples were measured following GB/T 1346-2011 [64] guidelines. The setting time of the sample was measured by the Vica instrument (Jingwei Test Instrument Co., LTD., Shijiazhuang, Hebei, China). The setting time included the initial setting time and the final setting time.

#### 2.2.3. Calorimetric Measurements

Heat of hydration was determined according to standard GB/T 12959-2008 [65]. Isothermal calorimetry was used in this study. TAM Air channel microcalorimeter (TA Instruments, Shanghai, China) was used to measure the hydration heat release performance of the sample, and the measurement method of external stirring was selected. First, 4 g of the sample was accurately weighed and placed in a plastic ampoule. After the machine showed baseline balance, 2 g deionized water was quickly injected into the ampoule. After two minutes of mixing, the cement was put into a micrometer together with the Abe bottle, the hydration process was carried out, and the heat was released. The hydration heat release was measured at 20 °C within 72 h from the beginning of hydration.

#### 2.2.4. Compressive Strength

Compressive strength was measured according to standard GB/T 17671-2021 [61] and EN 197-1:2000 [66]. The structural and mechanical properties of cement-based materials were evaluated by measuring the compressive strength at 3, 7, 28, 60, and 180 days. TYE-300D bending compressive testing machine (Jianyi Instrument Machinery Co., LTD., Wuxi, Jiangsu, China) was used to measure the compressive strength of cement mortar, and the loading rate was set at 1.6 kN/s. The compressive strength of the sample was calculated using the average value of the six tests to reduce the influence of errors.

#### 2.2.5. Thermogravimetry

After 3 days, 7 days, 28 days, and 60 days, small pieces of paste samples were ground with an agate mortar, and the resulting powder was sifted into 200-mesh screens. The powder was placed in an Al_2_O_3_ pan. TG analysis was performed using a thermogravimetric analyzer (azhan Testing Instrument Co., LTD., Nanjing, Jiangsu, China) and was performed in a nitrogen atmosphere. The specific parameters were as follows: the temperature range was 25–1000 °C and heating rate was 20 K/min.

#### 2.2.6. X-Ray Diffraction (XRD)

X-ray diffraction (XRD) was used to assess the mineral composition of the sample. Small clean pulp samples of 3, 7, 28, and 60 days were ground with an agate mortar, and the resulting powder was sifted into 200-mesh screens. The LC^3^ samples were analyzed qualitatively and quantitatively. The sample and zinc oxide were mixed and ground evenly according to the mass ratio of 9:1, and the mixture was made uniform after 30 min of grinding. Measurements were made using a copper target (CuKα), and the results were obtained from D8 Advance diffractometer (Bruker Spectral Instruments, Beijing, China). The instrument parameters were set to a scanning speed of 4.6°/min and a diffraction angle range of 5° to 70°.

#### 2.2.7. Volcanic Ash Availability Coefficient (PEC)

To better evaluate the effectiveness of volcanic ash in blends, volcanic ash availability coefficient (PEC) was used in this study. *PEC* (*x*) represents *x*% of the availability of volcanic ash used as an alternative to Portland cement in blended cement. The specific formula is shown in Formula (1) below.

(1) $$PEC\left(x\right)=\frac{f_{c}\left(Px\right)-\left(1-\frac{x}{100}\right)\times f_{c}(R)}{f_{c}(R)\times \frac{x}{100}}$$

Here, *fc* is the compressive strength, *Px* is a slurry prepared with a mixed cement containing *x*% volcanic ash as a substitute for Portland cement, *R* is a reference slurry prepared with Portland cement only, and *x* is the content of volcanic ash in the mixed cement measured in % by mass.

#### 2.2.8. Hydration Degree

In order to quantitatively study the hydration degree of cement and the relative content of formed hydration products under different clay-type LS and LP contents, XRF–Rietveld analysis was used to calculate the hydration degree at 3 days, 7 days, 28 days, and 60 days. The hydration degree of cement in each sample was calculated according to Formula (2).

(2) $$DoH\left(t\right)=1-\frac{\left(C_{3}S+C_{2}S+C_{3}A+C_{4}AF\right)\left(t\right)}{\left(C_{3}S+C_{2}S+C_{3}A+C_{4}AF\right)\left(t=0\right)}\;$$

#### 2.2.9. Nuclear Magnetic Resonance (NMR)

^29^Si NMR spectroscopy was performed with the Agilent-NMR-vnmrs600 spectrometer (Agilent Technologies, Inc., CA, USA). The spectra were processed using XPSPEAK41 and MESTER-C software. ^29^Si NMR provides valuable information related to the formation of C-S-H gels, the main hydration phase of cement grout.

#### 2.2.10. Scanning Electron Microscopy (SEM)

Scanning electron microscopy (SEM) is a kind of microscopic analysis technology that scans the surface of a sample by means of a high-energy electron beam and obtains the surface morphology and composition information of the sample by detecting the signals generated by the interactions between the electrons and the sample. In this study, N.sem450 scanning electron microscope (FEI Corporation, Hillsboro, OR, USA) was used, and its operating voltage was set at 3.00 kV.

#### 2.2.11. Mercury Intrusion Porosimetry (MIP)

The pore structure was analyzed by mercury injection porosity method (MicroActive AutoPore V 9600 2.03.00, Micromeritics Instrument Corporation, GA, USA). This is a technique for determining the pore size and distribution of a material based on the non-wettability of mercury. Since mercury is not wettable to most solid materials, an external force (usually pressure) is needed to overcome the surface tension and allow mercury to enter the pores of the material. As the applied pressure increases, mercury can enter smaller pores. By measuring the mercury content at different pressures, the corresponding pore size can be calculated. The technology has a pressure range of 0.10 to 61,000.00 pounds per square inch and a contact angle of 130°.

## 3. Results and Discussion

### 3.1. Setting Time and Water for Standard Consistency

**Figure 5:**
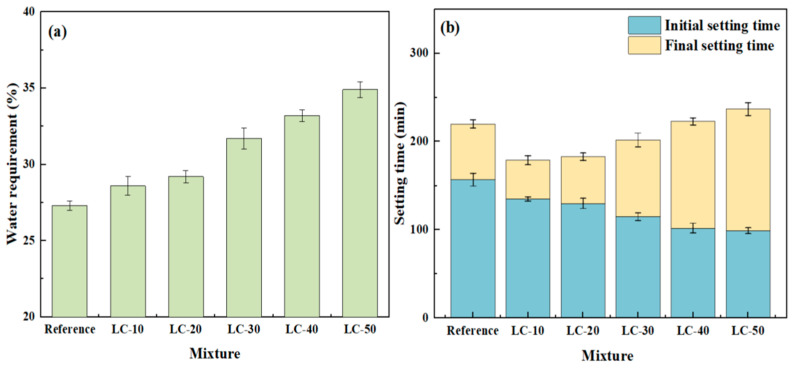
(**a**) Water consumption of standard consistency and (**b**) setting time for reference and LC.

The standard consistency, water consumption, and setting time of all samples are shown in Figure 5. The vertical error bar represents the standard deviation. As shown in Figure 5a, the reference sample’s standard consistency water demand was 27.3%. As the clay-type LS and LP content increased, the water demand rose accordingly, with LC-10, LC-20, LC-30, LC-40, and LC-50 requiring 28.6%, 29.2%, 31.7%, 33.2%, and 34.9%, respectively. According to the SEM test results for the clay-type LS, the increase in water consumption of standard consistency was mainly due to the layered structure of clay-type LS. When the cement was replaced by clay-type LS, the clay-type LS absorbed the free water in the system, which caused the free water in the slurry to decrease. Accordingly, the water consumption for the standard consistency also increased correspondingly.

To assess the influence of clay-type LS and LP on the setting time of cement-based materials, the setting times of samples with varying contents of clay-type LS and LP were measured, as illustrated in Figure 5b. The results indicated that as the clay-type LS and LP content increased, the initial setting time decreased, while the final setting time exhibited the opposite trend. There were two main reasons for the gradual decrease in initial setting time. On the one hand, the porous layered structure of LS would reduce the water in the system and reduce the water–solid ratio of the system in a certain period of time. On the other hand, clay-type LS and LP provided more nucleation sites for cement hydration and accelerated the cement hydration. The gradual increase in final setting time was mainly due to the co-action of the dilution effect and nucleation effect of the clay-type LS and LP. When the nuclear effect was bigger than the dilution effect, the final setting time became shorter, as shown by LC-10, LC-20, and LC-30. When the dilution effect was greater than the nucleation effect, the final setting time was extended, as shown by LC-40 and LC-50.

### 3.2. Heat of Hydration

**Figure 6:**
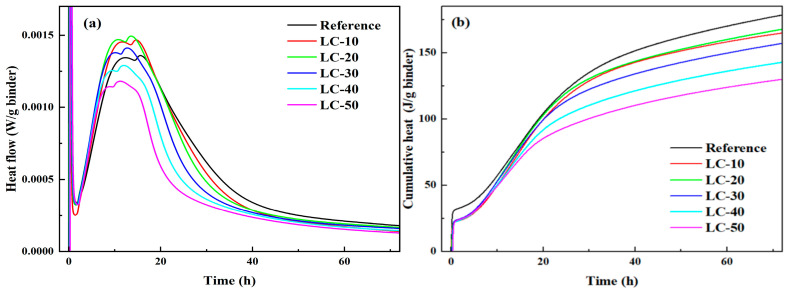
Heat evolution of LS normalized by weight of binder: (**a**) heat flow; (**b**) cumulative heat curve.

To investigate the impact of clay-type LS and LP on the hydration kinetics of cement-based materials, isothermal calorimetry was employed to analyze the hydration process, with the findings presented in Figure 6.

It can be seen from Figure 6a that the incorporation of clay-type LS significantly affected the early hydration of the cement. The difference in early hydration heat release between the LC^3^ cement and reference cement, especially within the first 10 min, may be due to a variety of factors, including mineral composition, hydration dynamics, and the role of water in the cement dissolution process. The clay-type LS had two functions in the early stage of hydration, a dilution effect and a nucleation effect. However, the induction period did not shorten, possibly due to the slow dissolution rate or the inhibition of the protective layer, which limited the hydration rate. The time to reach the maximum heat release peak was earlier than that of the baseline group. Moreover, the maximum exothermic peaks of LC-10, LC-20, and LC-30 were higher than those of the reference. The main reason for this phenomenon was that when LS replaced cement, its porous property provided more nucleation sites for cement hydration and accelerated the cement hydration. In addition, LP also had the effect of accelerating the hydration of cement. The nucleation effect of the clay-type LS played a dominant role in the system during this period. However, the peak value of the maximum exothermic peak of LC-40 and LC-50 was smaller than that of the benchmark group, mainly because the dilution effect was dominant. The result was in good agreement with that of the condensation time test.

Figure 6b shows that the order of cumulative heat release, from high to low, was reference, LC-20, LC-10, LC-30, LC-40, and LC-50, and the dilution effect of the reference was higher than that of the other samples. The reason why the cumulative heat release of LC-20 could exceed LC-10 was that clay-type LS and LP work together to accelerate the hydration of cement and increase the cumulative heat release.

### 3.3. Mechanical Properties

**Figure 7:**
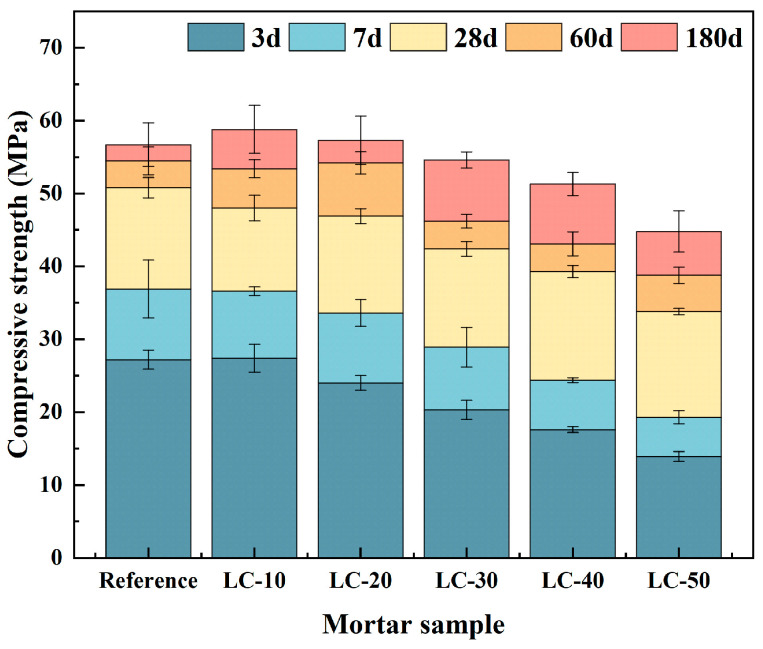
The compressive strength of the reference and LC.

To examine the effect of clay-type LS and LP on the mechanical performance of cement-based materials, the strength of samples with varying clay-type LS and LP contents was tested, as depicted in Figure 7. The vertical error bar represents the standard deviation.

The figure reveals that as the clay-type LS and LP content increased, the 3-day, 7-day, and 28-day hydration times of clay-type LS exhibited a steady decline. This phenomenon might be caused by the lower activity of the clay-type LS, so that the amount of volcanic ash reaction was less and it formed fewer hydration products. It mainly showed the dilution effect of clay-type LS. It is worth noting that, corresponding to the results of the water quantity of the standard consistency, the w/c increased with the higher amount of clay-type LS and LP incorporation. Too high a w/c led to a reduction in the strength of the cement paste. As the curing period extended, the quantity of hydration products in the system progressively rose. Therefore, with the increase in clay-type LS content at 60 days, the compressive strength first increased and then decreased. Among them, LC-20 had the highest compressive strength, with a compressive strength of 54.2 MPa, which was similar to the reference. However, LC-30, LC-40, and LC-50 were still lower than the reference, and clay-type LS still showed a dilution effect. With the increasing curing age, the amount of hydration products generated by volcanic ash reaction continued to increase. At 180 days, the compressive strengths of LC-10 and LC-20 exceeded that of the reference by 3.7% and 1.1%, respectively. The mechanical properties of LC-30 were comparable to the reference. Compared with the reference, the mechanical properties of LC-40 and LC-50 decreased by 9.5% and 21.0%, respectively. Based on the results of the mechanical properties test, the parameter of clay-type LS and LP should not be greater than 40%.

Based on the 180-day compressive strength data, it was found that the PEC of LC-10 to LC-50 was 1.56, 1.08, 0.81, 0.64, and 0.37, respectively. If 0 < *PEC* (*x*) < 1, the material being analyzed acts as volcanic ash in the blend with Portland cement. The higher the PEC value, the higher the pozzolanic property. If *PEC* (*x*) < 0, then the material cannot be considered volcanic ash and acts only as a filler. If *PEC* (*x*) > 1, it indicates that the availability of volcanic ash is higher than that of Portland cement, and there is a synergistic effect in the Portland cement–pozzoline–water system [67]. For LC-10 and LC-20, PEC was greater than 1, indicating a synergistic effect in the LC^3^ system. On the other hand, the effectiveness of LC-50 as volcanic ash in this mixture was very low. It acted more as filler, but not completely. Thus, the usability of the LC^3^ cement was proven.

### 3.4. XRD and Thermogravimetric Analysis

**Figure 8:**
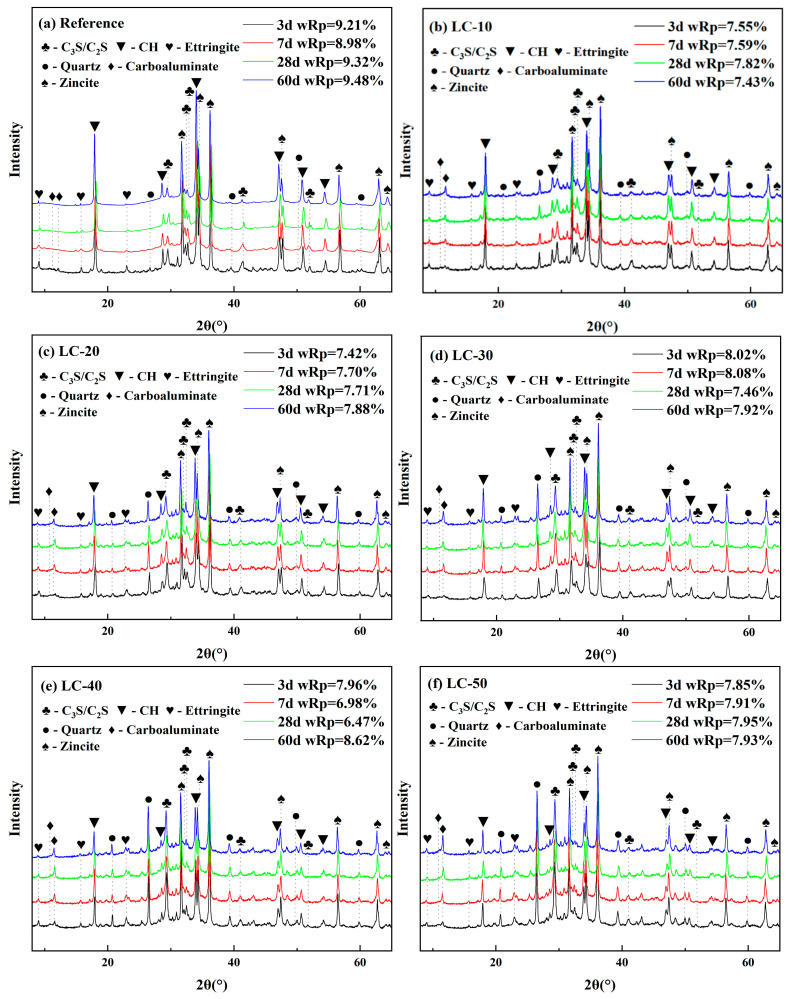
XRD spectra of reference and LC.

The hydration products of the system were analyzed by XRD, and the results are shown in Figure 8. The figure indicates that the primary mineral phases in the cement paste consisted of diffraction peaks corresponding to C_2_S, C_3_S, CH, CaCO_3_, SiO_2_, and other minerals. According to the results of the XRD quantitative test, no diffraction peak of calcium carboaluminate was found in the reference. When clay-type LS replaced cement, the carboaluminate was found in the system. With the increase in clay-type LS content and the extension in the curing age, the diffraction peak of carboaluminate became more and more obvious. This indicates that the aluminum phase in the clay-type LS was involved in the reaction of the hydration product with LP to form calcium carboaluminate. With the increase in the amount of clay-type LS and LP, the amount of calcium carboaluminate produced by the reaction increases correspondingly.

**Figure 9:**
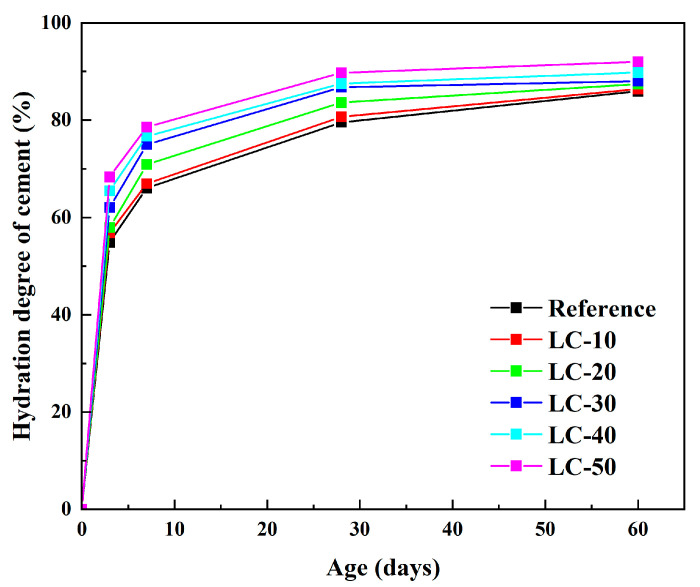
The hydration degree of the reference and LC.

The results of hydration degree are illustrated in Figure 9. The figure reveals that the hydration degree of cement increased as the curing period was extended. During the early hydration phase, the cement had already undergone significant hydration. Within the first 7 days, the hydration rate exceeded 50%. As the clay-type LS and LP content rose, the hydration rate of the cement showed a noticeable increase. However, as the curing age progressed, the hydration rate gradually slowed down. The main reason for this phenomenon is that the reaction of cement with water is a solid–liquid reaction. After reaching a certain age, the hydration products had wrapped the cement particles, affecting the water transport and slowing down the hydration rate of the cement.

**Figure 10:**
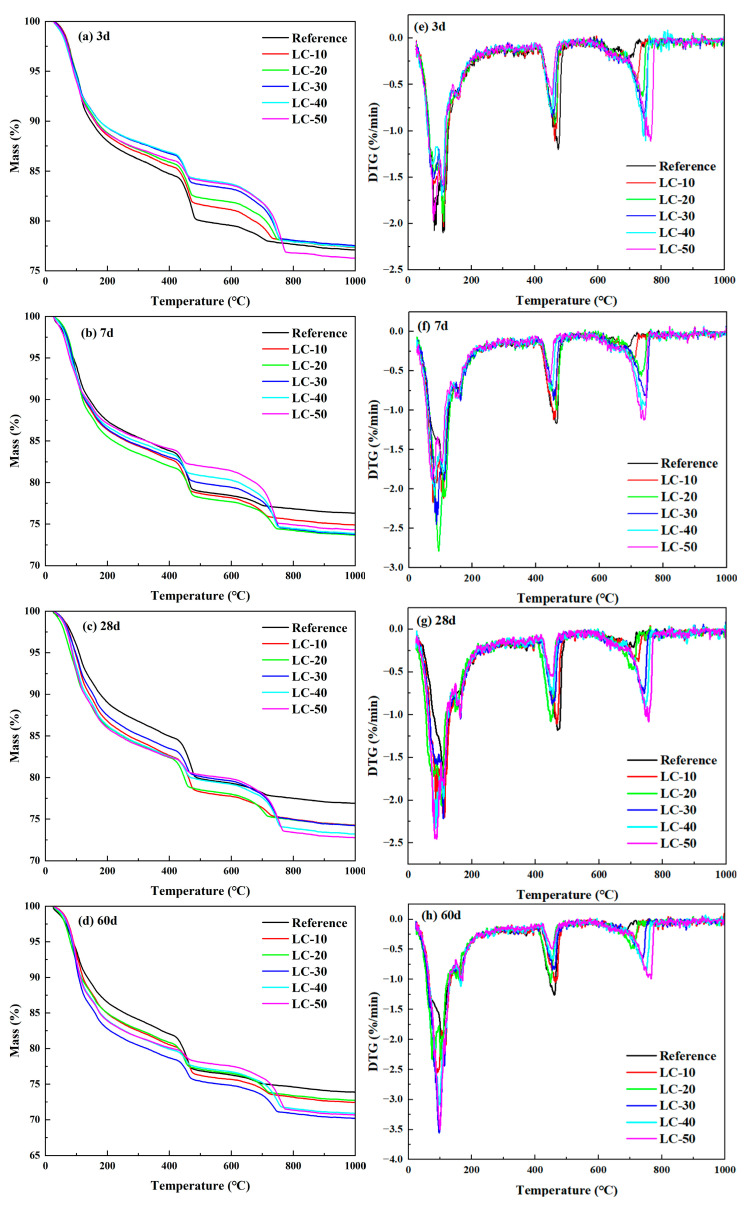
TG and DTG curve of the reference and LC.

To investigate the impact of clay-type LS and LP on the hydration products, thermal analysis was conducted to characterize the hydration products of the cement-based materials, with the results displayed in Figure 10. The figure indicates that the reference and LC samples exhibited similar trends, although variations in the mineral phase content resulted in differing magnitudes of mass change. Previous studies had shown that the mass loss on the DTG curve between 20 and 400 °C was mainly caused by the decomposition of C-(A)-S-H, AFt, and hemicarboaluminate/monocarboaluminate (Hc/Mc). The mass change within this temperature range was influenced by both the dilution effect and the volcanic ash effect of the clay-type LS and LP. Therefore, the quality loss was not significant with the variation in clay-type LS and LP content. The endothermic peak at 400–500 °C was the thermal decomposition of the hydration product CH. As the content of clay-type LS and LP increased, the exothermic peak of CH decomposition became weaker and weaker. This indicates that the CH content in the system gradually decreased, mainly due to the volcanic ash effect and dilution effect of clay-type LS and LP. In addition, a peak was found in the range of 600–800 °C, mainly attributed to the decomposition of CaCO_3_.

The mass loss of cement-based materials at 400–500 °C was quantitatively analyzed according to a DTG curve. The CH content in the cement-based materials at various curing ages was determined, with the results presented in Table 3. The CH content in the reference gradually increased with age. The CH content in the LC-10, LC-20, and LC-30 samples showed an increasing trend from 3 days to 28 days but a decreasing trend from 28 days to 60 days of age. The initial rise was attributed to the nucleation effect of the clay-type LS and LP, which accelerated cement hydration, producing additional CH to offset the CH consumed by the pozzolanic reaction. The later reduction was due to the consumption of CH in the volcanic ash reaction system of the clay-type LS, which generated more hydration products (C-(A)-S-H, Hc/Mc). It was observed that the CH content in LC-40 and LC-50 initially decreased, then increased, and subsequently decreased again over time. This trend was primarily attributed to the combined influence of the pozzolanic reaction and the nucleation effect of clay-type LS.

### 3.5. Nuclear Magnetic Resonance

**Figure 11:**
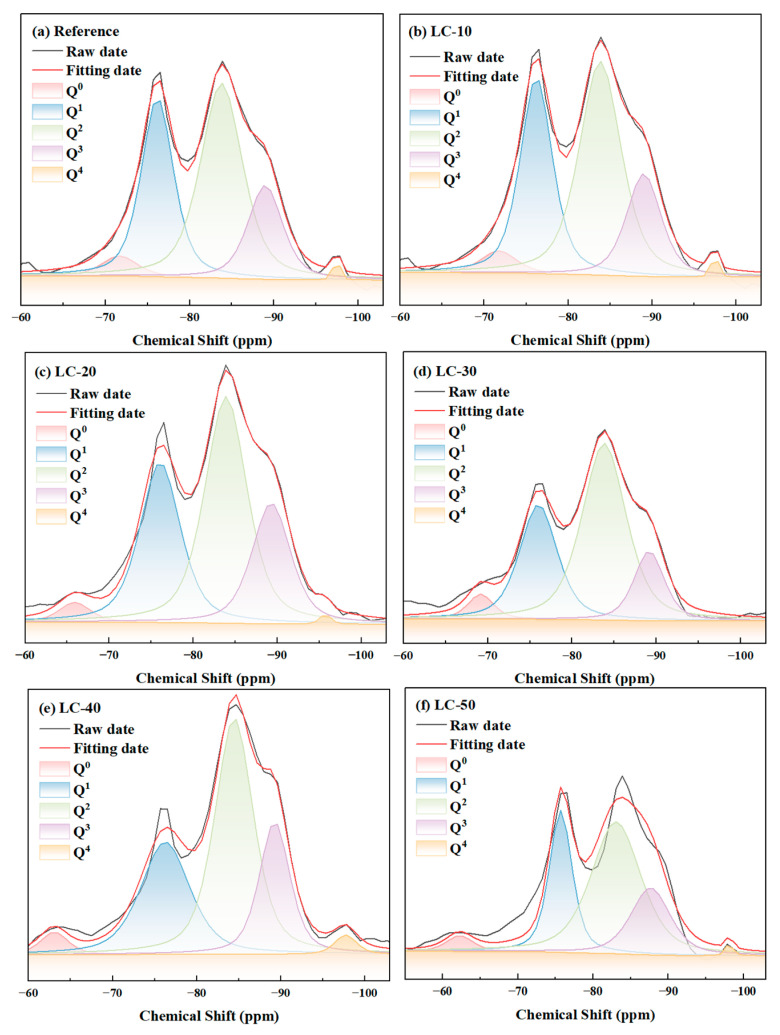
^29^Si NMR spectra of reference and LC.

To further investigate the impact of clay-type LS on the hydration products of cement-based materials, ^29^Si NMR analysis was conducted, and the findings are presented in Figure 11. According to previous studies [68], the Q^0^ peak corresponds to unhydrated cement. In this study, as the clay-type LS and LP content increased, the relative peak area of Q^0^ showed a gradual decline. Specifically, the Q^0^ relative peak area from LC-10 to LC-50 decreased from 25.04 to 16.65. This phenomenon was primarily caused by the combined influence of the dilution and nucleation effects of clay-type LS. In addition, the Q^3^ and Q^4^ values in samples containing clay-type LS were complex, which was related to the silicate tetrahedron of chalco-silicate glass and quartz in clay-type LS.

Q^1^ and Q^2^ were primarily associated with hydration products. In this research, the polymerization degree of hydration products and the average molecular chain length were determined, with the outcomes displayed in Table 4. In the reference, the average chain length and polymerization degree of C-S-H were 3.80 and 0.90, respectively. Furthermore, it was noted that the polymerization degree and average molecular chain length of the hydration products rose as the clay-type LS and LP content increased. Compared with the reference average, the C-S-H chain length of the LC samples grew by 27.63% to 59.21%, while the polymerization degree expanded by 57.78% to 125.56%. This indicates that the pozzolanic reaction and nucleation effect of the clay-type LS accelerated the cement hydration in the system.

### 3.6. Pore Structure Analysis

**Figure 12:**
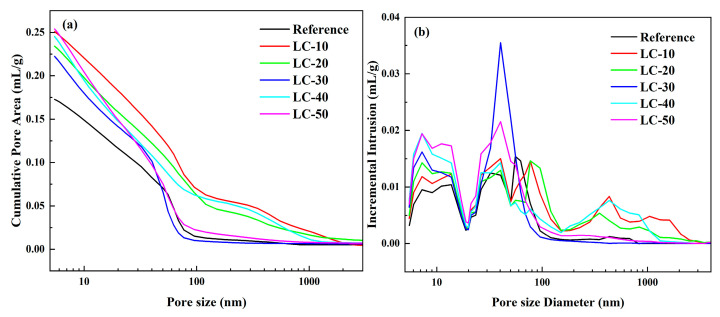
(**a**) The cumulative pore size and (**b**) pore size distribution of the reference and LC.

To investigate the impact of clay-type LS on the microstructure of cement-based materials, MIP was employed to analyze the pore structure, with the results illustrated in Figure 12. The data revealed that the particle size of all samples predominantly fell within the 3–100 nm range. The pore size distribution of the reference included some pores measuring 80–100 nm. However, after incorporating clay-type LS and LP, the number of 3–20 nm pores in the cement-based materials increased significantly. Combined with SEM results, it was found that this refinement was mainly due to the clay-type LS. The appropriate number of micropores could promote the hydration reaction and improve the late strength of the material. The pozzolanic reaction of clay-type LS consumed CH in the system and produced additional hydration products, thus filling the pores.

Table 5 presents the total porosity of cement-based materials with varying LS contents. With the increase in clay-type LS and LP, its porosity also increased to varying degrees. This was mainly because the early pozzolanic activity of clay-type LS was low, and more hydration products were not formed to make up for the reduction in total hydration products caused by its dilution effect. This result had a strong correlation with the results of the strength test.

### 3.7. Environmental Evaluation

In this study, LC^3^ cement was a novel low-carbon cement formulation, consisting of calcined clay-type LS, LP, and cement clinker. Its production process not only makes efficient use of industrial waste, but also significantly reduces carbon emissions. Environmental assessment of LC^3^ cement was carried out to analyze its environmental impact during raw material acquisition, production, use, and disposal. It was compared with traditional cement to provide a scientific basis for the popularization and application of low-carbon cement.

LC^3^ cement is compared with conventional cement from raw materials. The main component of traditional cement is Portland cement clinker, which requires a large amount of mineral resources such as limestone mining [49]. Cement clinker is the core raw material in traditional cement production, and its production process requires high-temperature calcination, high energy consumption, and carbon emissions. The use of clay-type LS in LC^3^ cement not only reduces waste generation, but also reduces raw material costs. At the same time, the demand for high-quality limestone is reduced, reducing the environmental pressure at the raw material acquisition stage.

During the service stage, the traditional cement concrete had relatively poor durability, and it was prone to cracking and water seepage, etc. It increases the resource consumption and environmental burden. The performance of LC^3^ cement is not inferior to traditional cement, and even has more advantages in some aspects. LC^3^ cement significantly enhanced the mechanical performance at later stages and optimized the void structure. LC^3^ cement can also be used to stabilize soil contaminated by heavy metals and reduce the migration of heavy metals [69,70].

Waste traditional cement concrete is often landfilled, occupying land resources and causing dust pollution. LC^3^ cement concrete also faces the problem of waste disposal. However, it contributed to reduced resource consumption and lower CO_2_ emissions during the production process. Even if it is landfilled, its environmental impact is less than that of traditional cement concrete. More importantly, LC^3^ cement concrete can be used as recycled aggregate for new construction projects to achieve resource recycling [71].

## 4. Conclusions

In this study, clay-type LS is taken as the research object. On the basis of clarifying the basic mineralogical characteristics, the optimum calcination temperature was determined by activating the activity of pozzolanic ash by high-temperature calcination. LC^3^ cement was prepared to provide a scientific basis for the green development and utilization of clay-based lithium resources. The characterization results of LC^3^ cement were compared with those of Portland cement. Among the conclusions of the analysis carried out, the following points should be highlighted:

1. At the initial stage of hydration, with the increase in clay-type LS and LP content, the compressive strength showed a gradually decreasing trend. This was mainly due to the dilution effect of the clay-type LS. However, proper incorporation of clay-type LS and LP had a positive effect on the development of compressive strength in the later period. At 180 days, the compressive strength of LC-10 and LC-20 exceeded that of the reference by 3.7% and 1.1%, respectively. The volcanic ash availability coefficient of LC-10 and LC-20 were both greater than 1, indicating that there was synergy in the LC^3^ system.
2. Proper incorporation of clay-type LS and LP could add additional hydration products, thus helping to fill large pores. In particular, the calcined clay-type LS reacted with calcium hydroxide to form C-A-S-H gel and amorphous aluminosilicates of aluminate hydrate.
3. From the point of view of low-carbon development, the optimum dosage of LC^3^ cement was studied by considering many factors such as mechanical properties, setting time, heat of hydration, and nuclear magnetic analysis. The studies showed that the optimal content of clay-type LS and LP should not exceed 30%.
4. LC^3^ cement partially replaced cement clinker with calcined clay-type LS and limestone, which significantly reduced CO_2_ emissions and energy consumption during the cement production. Its promotion and application strongly promoted the green and low-carbon transformation of the cement industry and provided important support for achieving the sustainable development goals.

## Figures and Tables

**Table 1:** The chemical composition of Cement I, LS, and LP (%).

	Na_2_O	MgO	Al_2_O_3_	SiO_2_	P_2_O_5_	SO_3_	K_2_O	CaO	TiO_2_	Cr_2_O_3_	Fe_2_O_3_	LOL
Cement I	0.58	0.86	4.78	19.19	0.11	3.31	0.55	65.8	0.51	-	3.99	0.32
LS	0.07	0.23	19.60	73.53	0.09	0.03	1.30	2.17	1.12	0.01	1.83	0.02
LP	-	2.17	0.11	0.23	-	0.05	-	53.40	-	-	0.03	44.01

**Table 2:** The mix ratio of samples (g).

	Cement I	LS	LP	Sand	Water
Reference	450	-	-	1350	225
LC-10	405	30	15	1350	225
LC-20	360	60	30	1350	225
LC-30	315	90	45	1350	225
LC-40	270	120	60	1350	225
LC-50	225	150	75	1350	225

**Table 3:** The content of CH in the samples (%).

Mixture	Reference	LC-10	LC-20	LC-30	LC-40	LC-50
3 days	19.32	15.99	14.68	12.70	11.10	8.63
7 days	20.19	17.06	15.54	12.74	10.89	8.55
28 days	21.01	18.01	15.91	13.85	11.59	8.72
60 days	21.34	17.68	15.42	13.44	10.65	8.30

**Table 4:** Peak positions and peak areas of reference and LC.

Mixture	Reference	LC-10	LC-20	LC-30	LC-40	LC-50
Q^0^	26.78	25.04	22.35	22.09	21.90	16.65
Q^1^	211.22	201.65	227.47	150.65	171.93	127.54
Q^2^	190.39	287.13	335.88	272.22	319.16	258.38
Q^3^	121.84	114.29	164.30	74.22	141.83	104.23
Q^4^	5.11	6.25	3.90	0.10	14.97	3.52
φ	3.80	4.85	4.95	5.61	5.71	6.05
PD	0.90	1.42	1.48	1.83	1.86	2.03

**Table 5:** The total porosity of samples with reference and LC.

Mixture	Ref.	LC-10	LC-20	LC-30	LC-40	LC-50
Total Porosity (%)	25.62	34.47	32.93	31.58	33.33	34.46

## Data Availability

The original contributions presented in this study are included in the article. Further inquiries can be directed to the corresponding author.
